# Assessing medical student documentation using simulated charts in emergency medicine

**DOI:** 10.1186/s12909-018-1314-z

**Published:** 2018-08-28

**Authors:** Wirachin Hoonpongsimanont, Irene Velarde, Christopher Gilani, Michael Louthan, Shahram Lotfipour

**Affiliations:** 1 Department of Emergency Medicine, University of California, Irvine, Irvine, CA USA; 2 Orange, USA

**Keywords:** Medical documentation, Medical students, Emergency medicine, Curriculum design

## Abstract

**Background:**

The 1995 Health Care Financing Administration (HCFA) guidelines stated that providers may only use the review of systems and past medical, family, social history in student documentation for billing purposes; therefore, many providers viewed the student documentation as an extraneous step and chose not to allow medical students to document patient visits. This workflow negatively affected medical student education in documentation skills. Although the negative impact on students’ documentation skills is obvious, areas of deficits are unknown. Understanding the area of deficits will benefit future curriculums to prepare prospective resident physicians for proper documentation. We aimed to assess areas of deficits in documentation of fourth-year medical students according to HCFA billing guidelines.

**Methods:**

We conducted a prospective study of fourth-year medical students’ simulated chart documentations at a United States medical school from May 2014 to May 2015. We evaluated students’ simulated charts from an online learning tool using simulated cases for completeness according to HCFA guidelines and analyzed data using descriptive statistics.

**Results:**

We found that 98.9% (*n* = 90) of the charts were downcoded. Of these charts, 33.0% (*n* = 30) had incomplete history of present illness, 90.1% (*n* = 82) had incomplete review of systems, 73.6% (*n* = 67) had incomplete past medical, family, social history and 88.8% (*n* = 80) had incomplete physical exams.

**Conclusion:**

New curriculum should include billing guideline information and emphasize the completeness of charts according to acuity.

## Background

Traditionally, the opportunity for medical students to assist with patient documentation has been an important part of their education. Participation in medical documentation allows medical students to develop the necessary skills of communicating with the patient and healthcare team and to apply and prioritize clinical information [1]. Association of American Medical Colleges and the Accreditation Council for Graduate Medical Education include documentation skills as one of their learning goals. United States Medical Licensing Examination also tests this skill on the Step II Clinical Skills Exam which every medical student must pass in order to graduate [2].

In the United States, the Centers for Medicare and Medicaid Services (CMS) use the HCFA Common Procedure Coding System for medical billing processes. The HCFA Common Procedure Coding System includes ICD-10 codes to report medical services and Current Procedural Terminology (CPT) Codes, which include patient type, setting of service and level of evaluation and management service performed. The three main areas, when choosing the appropriate code for evaluation and management services, are history, examination and medical decision making [3].

According to the 1995 Health Care Financing Administration (HCFA) guidelines, emergency department (ED) medical billing uses five CPT codes: codes 99,281–99,285, which are determined by the complexity of the ED visit and completeness of the medical documentation. CPT code 99281 is the least complex code and requires the least amount of chart documentation: only one component of history of present illness, a limited exam of one affected body area, and no requirements to document review of systems, and past medical, family, social history. The highest complexity code is 99285 which is typically used for admitted or transferred ED patients. The code 99285 requires providers to document at least four components of history of present illness, complete review of systems, all past medical, family, social history, and greater than eight organ systems in the physical exam section [4–7].

In an attempt to reduce incorrect billings, the HCFA released a new billing guideline that aggressively reinforces the billing rules. According to an Association of American Medical Colleges report, the U.S. does not consider medical students as billing providers, as they are not licensed yet, and mandate that their notes should not be included in the patient medical record. The CMS has strict regulations concerning which student documentation can be used for billing to decrease fraudulent billing practices. Specifically, a teaching physician may use review of systems and past medical, family, social history when documented by a medical student but all other information must be re-documented by the physician. Although these guidelines are specific to CMS patients, multiple specialties and institutions follow these guidelines with other patients [8, 9]. Because physicians cannot use medical student documentation for their actual documentation, the medical student documentation becomes an additional document for the physician to approve alongside their own documentations. This challenge makes the use of medical student documentation in real practice impractical [10, 11]. Many providers opted out from having their medical students perform any documentation at all.

Furthermore, correct coding practices influence physician reimbursement. After the release of the 1995 HCFA guidelines, 64% of clerkship directors voiced concerns that the guidelines would adversely affect the quality of medical student educational programs [11]. While medical educators proposed alternative methods to teach medical documentation techniques, such as using separate notes for medical students or creating a special secured environment for medical students to document in case if auditing occurs, healthcare providers raised concerns on potential losses in productivity due to work duplication [1]. A survey of Emergency Medicine (EM) residents revealed that only 4% of them were confident in their ability to document for billing and coding which reflects inadequate training in medical documentation in the medical school curriculum [10]. A recent study of coding practices in pediatric radiology shows that “appropriate documentation, informed by knowledge of coding, billing and reimbursement fundamentals” affect reimbursement and payment of services. Similar results were reported for a vascular surgery procedural reimbursement study [12, 13].

This study aims to identify areas of deficits in medical documentation by assessing simulated medical charts from fourth-year medical students during their EM clerkship.

## Methods

### Study design and setting

We conducted a prospective, chart review study to evaluate the quality of simulated medical student chart documentations at an U.S. medical school from May 2014 to May 2015. Researchers collected the simulated chart documentations that were completed by all fourth-year medical students who participated in the EM clerkship during the study period. This resulted in a sample size of 104 chart documentations. The documentations were automatically sent to the clerkship director as a part of the curriculum requirement. The clerkship director de-identified all documentations prior to distribution to the research team and any data collection. This study was approved by the Institutional Review Board under the exempt category as no personal information was collected.

### Study protocol

As part of the EM clerkship at our institution, medical students completed an online patient encounter and submitted a simulated patient documentation as part of their EM clerkship curriculum requirement. The students used the Digital Instruction in Emergency Medicine, an online program developed by the Clerkship Director of Emergency Medicine group, to enhance the medical students’ EM experience. Each student chose one of three available online, written scenarios to practice his/her clinical skills (including history taking and physical exam, medical assessment and plan) and then documented their findings and treatments in this online platform. Once the medical students completed the case and documentation, the simulated charts were automatically sent to the EM clerkship director for review. This assignment allowed medical students to practice their documentation skills on simulated patient encounters. After the clerkship director de-identified all simulated documentations, a coding and collection specialist who is certified by the American Academy of Professional Coders (AAPC) analyzed the charts for their completeness and compliance with the 1995 HCFA guidelines using a billing audit tool [7]. Only charts with the highest complexity, code of 99,285, were sent to the coding specialist to review. The coding specialist assessed the availability of each portion of the simulated charts including patient’s chief complaint; history of present illness; review of systems; past medical, family, social history; and physical exam. The specialist then provided the suitable billing codes according to the HCFA guidelines. The coding specialist reviewed only the simulated charts and had no access to additional information regarding the simulated patient cases and medical students. For any charts that had suitable billing codes less than 99,285 (99281–99,284), we referred to them as a “downcoded” chart and recorded the reason for downcoding on an excel spreadsheet.

### Outcome variables and data analysis

We reported the percentages of the downcoded simulated charts and percentages of absence or incompleteness of the charts in each category: chief complaint; history of present illness; review of systems; past medical, family, social history; and physical exam. We used descriptive statistics to analyze the data.

## Results

Ninety charts out of the ninety-one simulated charts with a code of 99,285 were downcoded, indicating incomplete documentation by the medical students (98.9%, 95% CI: 94.0–99.9%).

The most common cause of downcoding was an incomplete review of systems. Review of systems was incomplete in 90.1% (*n* = 82, 95% CI: 82.1–95.4%) of the charts.

Physical exam was the second most incomplete section in the studied charts. While a general multisystem (≥10 organ systems) or complete single organ exam was required in the simulated charts, 88.8% (*n* = 80, 95% CI: 79.4–93.8%) of charts did not meet the expectation.)

Documentation of past medical, family, social history was required in the studied charts. Past medical, family, social history was incomplete in 73.6% (*n* = 67, 95% CI: 63.3–82.3%) of the charts. History of present illness was incomplete in 33.0% (*n* = 30, 95% CI: 23.5–43.6%) of the charts.

Past medical history was completed in 82.4% (*n* = 75, 95% CI: 73.0–89.6%) of the charts. Only 22.0% (*n* = 20, 95% CI: 14.0–31.9%) of the charts had a complete social history and only 2.2% (*n* = 2, 95% CI: 0.3–7.7%) had a complete family history (Table 1).

**Table 1:** Percentages of complete documentation in each section of simulated charts

Section	Complete (%)
History of present illness	
Location	103 (100%)
Quality	67 (64.4%)
Severity	91 (87.5%)
Duration	72 (69.2%)
Timing	53 (50.1%)
Context	22 (21.1%)
Modifying factors	43 (41.3%)
Associated signs and symptoms	5 (4.8%)
Review of systems	
Problem Focused (0)	8 (7.6%)
Expanded Problem (1)	14 (13.4%)
Detailed (2–9)	81 (77.8%)
Comprehensive (≥10)	0 (0%)
Past medical, family, social history	
Past Medical History	82 (78.8%)
Family History	2 (1.9%)
Social History	20 (19.2%)
Physical exam	
Limited exam of affected Body Area or Organ System (1)	8 (7.6%)
Limited exam of affected Body Area(s) and other related organ system (2–4)	44 (42.3%)
Extended exam of Body Area(s) and other symptomatic related Organ System(s) (5–7)	41 (39.7%)
General multi-system exam or complete single Organ System exam (≥8)	10 (9.6%)

**Fig. 1:**
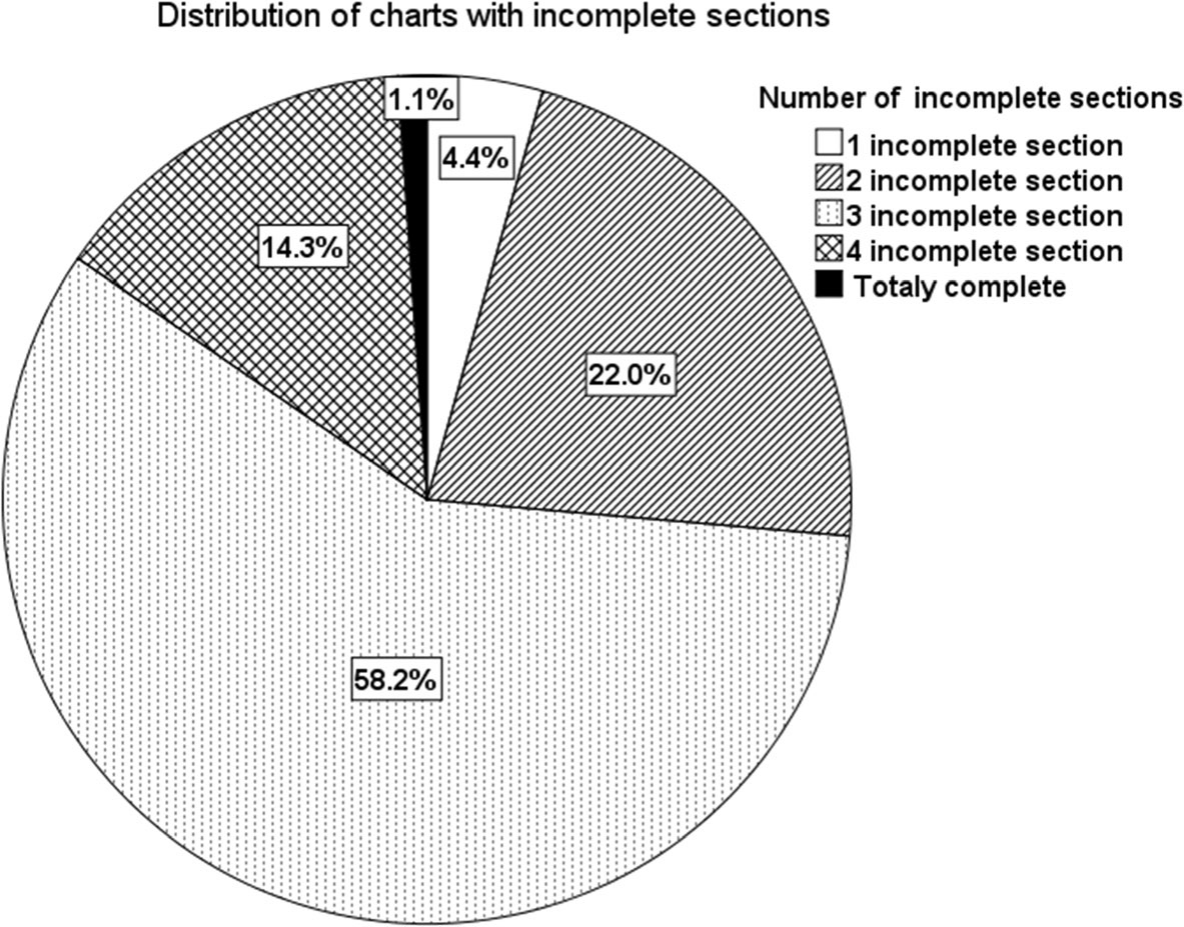
Distribution of charts with incomplete sections

**Fig. 2:**
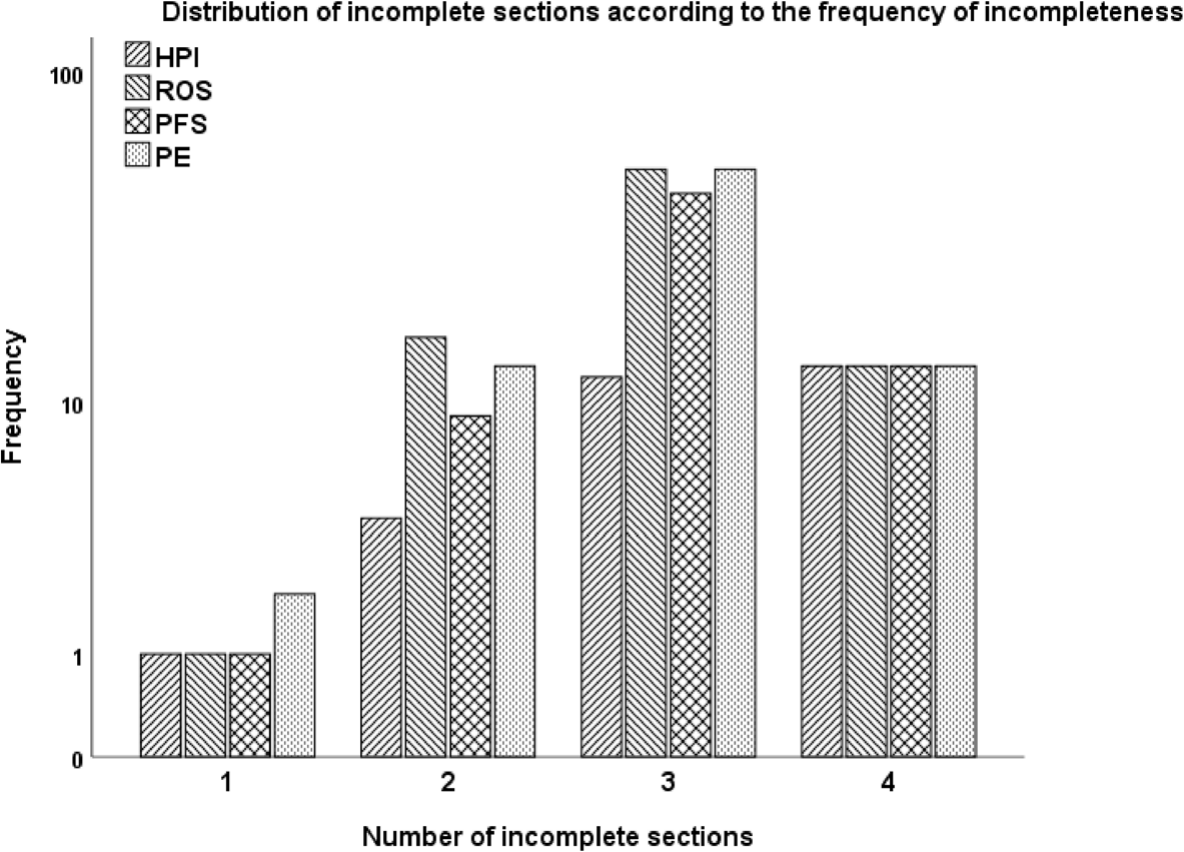
Distribution of incomplete sections according to the frequency of incompleteness. Abbreviations: *HPI* history of present illness, *ROS* review of systems, *PFS* past medical, family and social history, *PE* physical exam

Among the charts with downcoding, only 4.4% (*n* = 4, 95% CI: 1.2–10.9%) had just one incomplete section (Figs. 1 and 2).

## Discussion

We found that the majority of simulated charts were downcoded due to multiple incomplete sections in the charts. Review of systems, physical exam and past medical, family, social history were incomplete more often than other sections and history of present illness was rarely the cause of downcoding. Our findings reflect that medical student documentation was suboptimal for billing purposes due to the incompleteness in documentation. Many reasons could explain the findings including insufficient knowledge in chart billing. With these findings, educators should incorporate medical billing guidelines into their teaching curriculums and encourage medical students to practice documentation for both medical and billing purposes.

Although billing errors are typically seen in medical student documentations, physicians may also inaccurately bill for services or have trouble with understanding the documentation, coding, and billing rules as illustrated in a qualitative interview study of 32 family physicians [14]. One pilot study states that the most beneficial method to ensure accurate billing and coding techniques is to have physicians teach these skills as part of a medical school curriculum [15].

While many recommendations on how to prepare medical students for medical documentation according to the 1995 HCFA guidelines have been introduced to educators, only a few studies evaluated the utilization and effectiveness of these recommendations [9, 11]. At our institution, the students learn to document patient charts using their standardized patient encounters. There is a formal lecture about medical documentation delivered at the beginning of their first year. The students go through multiple simulated cases with standardized patients. The students submitted their medical documentations on Word document. Faculty from various specialties including family medicine, pediatrics and EM review the documents and provide feedback to students individually. A nursing training program in Korea used a mobile application to allow students to access an academic electronic medical record, a separate space for students to practice the documentation. The researchers concluded that this strategy provided students with more opportunities to practice on medical documentation [16]. A study in 1999 reviewed a self-directed learning program [17]. This program taught medical students about HCFA guidelines and medical documentation, then the students would document an example case. Afterward, the teaching physicians reviewed the documentations and provided feedback. While students indicated that this was effective in teaching medical documentation skills, teaching physicians stated that this method was not time efficient. Most medical schools do not appear to have formalized training in medical documentation for students to fit billing purposes prior to their clinical clerkships. However, many organizations including the AAPC and Coding Network offer training courses in documentation for healthcare professionals. Using these courses in conjunction with the current curriculum can enhance medical documentation skills in medical students.

Lack of formal training in medical documentation continues into various residency programs. Many residents felt that their documentation skills were inadequate [11]. A survey of pediatric residents showed that residents valued billing and coding skills, but they did not have adequate knowledge [18]. This sentiment is echoed across specialties including general surgery, family medicine and EM [19–21]. EM residents reported that the most common teaching method in medical documentation was informal teaching even though they found that formal feedback was the most helpful strategy [21]. We suggest that formal feedback could be given by the billing department, or medical students’ and residents’ charts could be sampled for review semi-annually or quarterly to minimize workload for teaching physicians. In addition, EM residents listed a lack of time as the greatest factor to limit proper education in documentation and coding strategies [21]. Residency programs could arrange the training courses prior to the official training year i.e. during resident boot camps. Many residency programs offer intern boot camps, but the course mainly focuses on procedures and simulated patient encounters. The training content should cover the current billing guidelines and emphasize the necessity of completing review of systems, physical exam; and past medical, family, social history.

Our study evaluated simulated charts that were created by medical students based on the information they acquired during the simulated encounters. Medical students’ documentation could be limited, causing downcoded charts, due to their incompetence in history taking skills. Furthermore, because the students were aware that their documentation would not have any effect on their clinical outcomes and/or medical liability, it is possible that they performed minimally to complete the simulated charts. This could have contributed to the incomplete documentation issues we found. Our study also lacked inter-rater reliability. We had access to only one coding specialist in the ED. The assessment by another coding specialist would decrease the bias or could generate different outcomes. Although, the training for billing and coding are quite consistent among the specialists; therefore, the effect of the second coding specialist is uncertain. Finally, our study focused on the compliance to billing guidelines, which may not correlate with proficiency in medical documentation for patient care.

## Conclusions

Majority of simulated medical student documentations were incomplete, especially in review of systems; physical exam; and past medical, family, social history, reflecting suboptimal coding and billing. Medical student educators and medical student governed organizations must emphasize on chart completeness. Billing guideline information and formal feedback on medical documentation should be mandatory in future curriculums.

## Abbreviations

AAPC: American Academy of Professional Coders
CMS: Centers for Medicare and Medicaid Services
CPT: Current Procedural Terminology
ED: Emergency Department
EM: Emergency Medicine
HCFA: Health Care Financing Administration
